# The development of human social learning across seven societies

**DOI:** 10.1038/s41467-018-04468-2

**Published:** 2018-05-25

**Authors:** Edwin J. C. van Leeuwen, Emma Cohen, Emma Collier-Baker, Christian J. Rapold, Marie Schäfer, Sebastian Schütte, Daniel B. M. Haun

**Affiliations:** 1 University of St Andrews, Westburn Lane, St Andrews, KY16 9JP Scotland; 2 Max Planck Institute for Psycholinguistics, Wundtlaan 1, 6500 AH Nijmegen, The Netherlands; 3 Institute of Cognitive and Evolutionary Anthropology, School of Anthropology and Museum Ethnography, University of Oxford, 51/53 Banbury Road, Oxford, OX2 6PE UK; 4 Wadham College, Parks Road, Oxford, OX1 3PN UK; 5 Forest, Nature and Environment Aceh, Banda Aceh, 23249 Indonesia; 6 School of Psychology, University of Queensland, St Lucia, QLD 4072 Australia; 7 Department of General and Comparative Linguistics, University of Regensburg, 93053, Regensburg, Germany; 8 Max Planck Institute for Evolutionary Anthropology, Deutscher Platz 6, 04103 Leipzig, Germany; 9 Leipzig Research Centre for Early Child Development & Department for Early Child Development and Culture, Faculty of Education, Leipzig University, Jahnallee 59, 04109 Leipzig, Germany

## Abstract

Social information use is a pivotal characteristic of the human species. Avoiding the cost of individual exploration, social learning confers substantial fitness benefits under a wide variety of environmental conditions, especially when the process is governed by biases toward relative superiority (e.g., experts, the majority). Here, we examine the development of social information use in children aged 4–14 years (*n* = 605) across seven societies in a standardised social learning task. We measured two key aspects of social information use: general reliance on social information and majority preference. We show that the extent to which children rely on social information depends on children’s cultural background. The extent of children’s majority preference also varies cross-culturally, but in contrast to social information use, the ontogeny of majority preference follows a U-shaped trajectory across all societies. Our results demonstrate both cultural continuity and diversity in the realm of human social learning.

## Abstract (web summary)

Social learning is a crucial human ability. Here, the authors examined children in 7 cultures and show that children’s reliance on social information and their preference to follow the majority vary across societies. However, the ontogeny of majority preference follows the same, U-shaped pattern across all societies.

## Introduction

Social information use is a crucial factor in the biological success of the human species^1^. The ability to capitalise on information obtained by others equips humans with a means to circumvent threats inherent to trial-and-error learning^2^. Moreover, humans excel in successively implementing socially-learned behaviours—also known as cumulative culture^3^ or the ratchet effect^4^—a process which leads, in the course of evolution, to increasingly adaptive solutions to environmental challenges^3^.

Individual exploration is costly compared to social learning: while the former requires time and effort dedicated to trial and error, the latter benefits from the investments of the social model. Moreover, individual exploration comes with comparably high risks, e.g., attempting  new strategies or uncovering new grounds is relatively prone to injuries and/or conflict. Hence, under such circumstances, social information use can be a fitness-increasing strategy^2^. Its expected payoff, however, depends on the way social learning is applied. If random conspecifics are chosen as models to learn from, social learning is unproductive. If skilful experts are copied, on the other hand, social learning becomes highly adaptive^5^. The challenge for the social learner lies in optimally identifying social models, especially given that quality is not always readily observable. For this reason, proxies of quality, like the majority vote^5–7^ or social status^8^, can be relevant heuristics. In particular, the strategy to side with the majority has received special attention because of its pervasive presence in numerous forms of human interaction^9,10^, and its direct relevance for understanding patterns of cultural evolution, given its potential to increase within-group homogeneity and between-group heterogeneity^2,7^.

Research with children in Western, industrialised societies has revealed potent social information use in general and majority preference in particular at an early age. For example, 14-month-old children already use social information rationally^11^. Children at 4 years of age even flexibly choose when to imitate others or innovate novel solutions^12^, the kind of advanced social information use precipitating adaptive learning and cumulative culture^13^. Selective sensitivity to the majority was reported for 2-year-old German children in a simple decision-making task similar to the one used in the current study^14^. Children watched four familiar, same age, same gender peers demonstrating the use of an unknown machine. Three peers demonstrated variant A once each, one peer demonstrated variant B three times. Two-year-old German children in this study preferentially copied the majority strategy^14^. Furthermore, underscoring the fundamental and adaptive nature of this learning bias, chimpanzees in the same study design also preferentially copied the majority^14^. In another study, 3–6-year-old American (United States) children were required to copy a necklace-making demonstration from video. Children showed greater imitative fidelity after watching two identical demonstrations by two different adults than after watching the same individual twice^15^. The developmental trajectory of these social learning phenomena, however, is less clear, since studies typically focus on a limited age-range. Notable exceptions are findings on increased over-imitation—i.e., copying both task-relevant and task-irrelevant aspects of a social demonstration—in 5–6-year-old children compared to 3–4-year-old children in the United Kingdom^16^, and above-chance selectivity in social learning strategies in 3-year olds developing into adaptive reliance on majority information in 7-year olds in the United States^17^. These studies, however, beg the question of whether the obtained results represent human universals or behavioural trends specific to Western, educated, industrialised, rich and democratic (i.e., WEIRD) societies^18^.

Cross-cultural studies on children’s social information use, including non-WEIRD societies, are scarce, and, to our knowledge, have not compared ontogenetic trends across societies and are usually limited to single comparisons between two societies. Provisionally, these studies indicate that culture-specific parenting styles yield cultural differences in children’s engagement in social learning situations and their proclivity to learn socially in general^19–21^. For instance, children raised in traditional Mayan family contexts, with explicit focus on registering ongoing social events, were found to pay attention to unaddressed social interactions (i.e., third-party attention) more markedly than European-American children^19^. On the other hand, in a practical task eliciting social learning, the preference to imitate in a conventional ('everyone always does it like this') rather than instrumental ('I am going to make a necklace') context was shared by two starkly different societies^20^. For children’s tendency to over-imitate, both cultural continuity^22^ and diversity^23^ have been reported.

Here, we set out to study human social learning across a range of cultures and ages. Our main goal was to elucidate, for the first time, whether reliance on social information develops in cross-culturally similar or variable patterns. Only by means of a cross-cultural comparison of developmental trajectories across a significant age span will we be able to identify patterns relevant to distinguishing universal from culture-specific socialisation processes^21,24,25^.

We administered a variant of a validated social learning task^14^ to children in societies spanning four continents, leading to a final sample of *n* = 605 children from 4 to 14 years old. Children were shown a video in which four German children demonstrated how to use a 'choice-box'^14^, which consisted of three differently coloured pipes and an automatic toy dispenser. Three demonstrators used one particular pipe, each throwing in one ball and receiving one toy per insertion, sequentially. One additional demonstrator used one of the two other pipes three times in a row, also receiving one toy per insertion. The majority (the three peers) and minority (the single peer) demonstrations were counterbalanced for order and across the three pipes. Subsequently, the child received one ball to use on the 'choice-box', after which we scored whether the child followed the majority, the minority, or used the third, non-demonstrated option (henceforth: 'innovation').

We report that while we find cross-cultural variation in both children’s reliance on social information and majority preference, only the ontogenetic trajectory of majority preference recurs across cultural contexts: The youngest and oldest children in our sample followed the majority most reliably across all societies. This cross-culturally recurrent developmental U-shape may reflect children’s changing capacities and motivations with respect to perceiving the majority as a valuable source of information, and as a potent force in shaping social relationships.

## Results

### The ontogeny of social information reliance across cultures

**Fig. 1:**
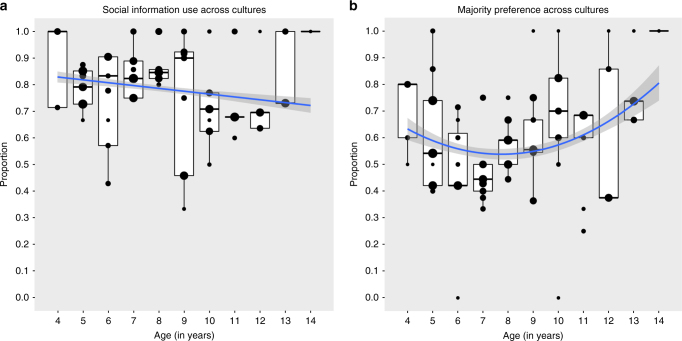
The ontogeny of social learning in human children. Depicted are **a** children’s reliance on social information (vs. innovation), and **b** majority (vs. minority) preference, across cultures. Blue lines represent ontogenetic trajectories across cultures; shaded area around the blue lines represent 95% confidence intervals. Medians are represented by the bold, horizontal lines within the boxes. The boxes represent the interquartile range (IQR), the vertical lines attached to the boxes represent Q1–1.5 IQR (lower) and Q3 + 1.5 IQR (upper). Dot size in **a** is proportional to the number of observations in ratio 1:5, with minimum and maximum number of observations per dot being 1 and 33, respectively. Dot size in **b** is proportional to the number of observations in ratio 1:5, with minimum and maximum number of observations per dot being 1 and 24, respectively

**Fig. 2:**
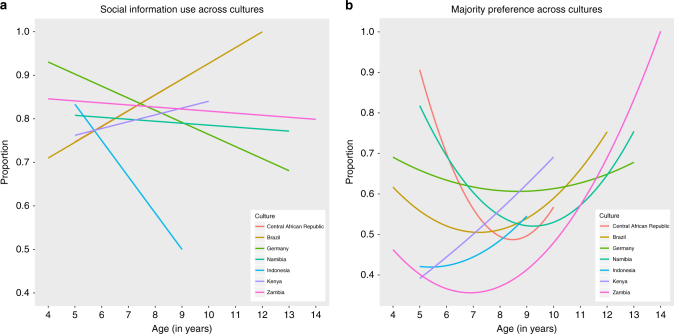
Culture-specific ontogenies of children’s social learning. Depicted are **a** children’s reliance on social information (vs. innovation), and **b** majority (vs. minority) preference, for each sampled culture. In **a**, *n* = 605, cultural diversity in social information use is represented by the differently valenced slopes across age (the slopes of Brazil and the Central African Republic in panel **a** almost perfectly overlap, see Supplementary Figure 1). In **b**, *n* = 475, cultural homogeneity in majority preference can be inferred from the similar U-shaped trajectories across age

Across cultures, we found that children’s reliance on social information (vs. innovation) slightly decreased with age (likelihood ratio test (LRT) age: *χ*^2^ = 3.99, df = 1, *n* = 605, *p* = 0.042, estimate ± SD = −0.22 ± 0.11; Fig. 1a). However, the ontogenetic trajectory of social information reliance varied substantially across cultures (LRT culture/age interaction: *χ*^2^ = 16.7, df = 6, *p* = 0.010, Fig. 2a and Supplementary Fig. 1). The nature of this age-effect difference was linear, not non-monotonous (LRT culture/age^2^: *χ*^2^ = 4.40, df = 6, *p* = 0.622). Across cultures, boys were 1.41 times more likely to rely on social information than girls were (LRT main effect sex: *χ*^2^ = 2.9, df = 1, *p* = 0.088). The order of demonstrations (majority and minority) did not significantly affect children’s inclination to use social information over innovating (LRT main effect order: *χ*^2^ = 1.70, df = 1, *p* = 0.192).

### The ontogeny of majority preference across cultures

Across cultures, the ontogenetic trajectory of the majority (vs. minority) preference showed a U-shaped pattern with both the relatively young and old children following the majority most markedly (LRT age^2^: *χ*^2^ = 4.38, df = 1, *n* = 475, *p* = 0.036; Fig. 1b). Comparing cultures, there was no evidence for cross-cultural heterogeneity in the U-shaped ontogenetic pattern for the majority preference (LRT culture/age^2^ interaction: *χ*^2^ = 3.98, df = 6, *p* = 0.679; Fig. 2b and Supplementary Fig. 2). Collapsed over ontogeny, societies did not differ in their proclivities to follow the majority (*χ*^2^ = 6.92, df = 6, *p* = 0.329). Again, boys tended to follow the majority more than girls did (*χ*^2^ = 3.30, df = 1, *p* = 0.082), with the odds for boys being 1.42 times larger than for girls. When the majority was demonstrated to the subjects first (and thus the minority second), the odds for children to follow the majority were 4.55 times larger than when the majority was demonstrated second (*χ*^2^ = 58.72, df = 1, *p* < 0.001). This pattern is suggestive of a primacy effect^26^. Regardless, by testing all variables in the same model, this effect was controlled for when gauging the impact of culture and age on majority preference.

## Discussion

Although the social demonstrations conferred adaptive information to the children, i.e., obtaining the reward is contingent on entering the ball into a (specific) hole of the apparatus, they did not become more reliant on social information with increasing age in all societies. Indeed, reliance on social information even tended to decrease with age in some societies, most pronouncedly in Germany and Indonesia. Such cultural differences spawn new lines of investigation into the dynamics of human social learning. For instance, future research with a selected set of contrasting cultures might examine whether a relatively high socio-economic standard (Germany and Indonesia (Jakarta) in our sample) might buffer individuals against potential costs incurred by individual exploration^2^ and consequently induce innovation. In summary, the present finding supports the notion that the tendency to rely on social information is not an ingrained mechanism in the developing human mind, but is malleable to cross-culturally variable circumstance^27^.

Children who readily relied on social information tended to prefer the majority mostly at a relatively young and old age, resulting in a U-shaped developmental trajectory. The U-shaped developmental curve is a recurrent phenomenon in developmental psychology^28^. Its emergence is typically explained by incremental learning becoming more complex (due to a demanding interplay between developing capacities), before it becomes more efficient. For instance, the ability to recognise gesture-referent contingencies in toddlers seems to decline first between 18–24 months of age, after which it reinstates at (latest at) the age of 4 years. This pattern has been explained in terms of an increasing appreciation of communicative conventions: early flexible use of arbitrary label-forms is followed by relative rigidity regarding object labels, after which children begin to understand the symbolic representation of language 'beyond the initial phase of using individual symbols to represent ideas in a one-to-one fashion'^29^. The cross-culturally recurrent U-shaped development of the majority bias is a new finding that speaks to the universal affordances of crowds^2,7,17^. Here, we envision the U-shape to emerge through a rudimentary majority bias early in development^14^, followed by increasing complexity of often-opposing social learning biases causing noise in decision-making, after which children begin to understand the contingency between proxy (i.e., the majority) and associated benefits, which enables more rational deliberation taking into account individuals’ aim to make correct decisions (i.e., informational conformity) or avoid social repercussions for being different (i.e., social conformity). In line with this conjecture, a recent study indicated that the majority bias guides decision-making differently in 3–4-year-old vs. 7-year-old American (United States) children, with the older children seemingly better able to understand relative majorities as useful proxies for high-quality information than their younger counterparts^17^. Additionally, changing social motivations could result in a variable tendency to follow the majority across ontogeny: Initial spontaneous, non-rational intuitions to follow the majority^30^ are succeeded by a phase of egocentrism resulting in relative indifference towards the majority, followed by deliberate, strategic majority choice^17^, based on the growing realisation of the social consequences of deviating. Similar ontogenetic patterns have previously been shown for prosocial tendencies^31^.

Lastly, the sex differences trend in social information use, especially with respect to the majority preference: Our finding that boys tended to follow the majority more markedly than girls did contrasts with existing literature on conformity sensu the Asch studies^32^. Within this seminal conformity framework, females (both juveniles and adults) are found to acquiesce to the majority more than males^30,33^, which has been linked to lower levels of self-reported confidence in one’s own judgement^34^. Note, however, the distinction between conforming against better knowledge and conforming in order to obtain a new behavioural variant. While the former is associated with social pressure and with the aforementioned sex bias, the latter does not require the forfeiting of an established notion or preference and is, as such, more relevantly linked to (adaptive) decision-making^17,35^. Importantly, the latter conformity type has, to our knowledge, not been investigated with a focus on sex differences. Hence, our study provides a first indication that boys are more biased towards following the majority in adopting novel behaviours than girls are.

Our findings uniquely evidence both the cultural malleability and cross-culturally recurrent features of the developing human mind. As explanation for finding both cultural variation and continuity in the realm of human social learning, we suggest that the extent to which social information is relied upon might be strongly influenced by society-specific affordances, while the specific bias to prefer the majority might emerge from more generic principles of homophily^36^ and optimal decision-making^2,7,14^. For instance, societies could differ in the extent to which potential costs of individual exploration (e.g., in terms of time and effort)^2^ can be buffered (e.g., by social security), which would affect reliance on social information, but not the relative superiority (in terms of informational value) of the majority over the minority. The systematic titration of the impact of societies’ characteristics on social information use is an exciting avenue for future research and could provide important insights into the socio-ecological correlates of the emergence of human culture.

An alternative explanation of our findings could be sought in differential responding to Caucasian model demonstrations by children from different cultural backgrounds. Caucasian children are frequently attributed with high status among peers across societies^37^. However, the cultural variability in social information use (Fig. 1a) is not consistent with such an ethnic bias, as not only German children showed a decreasing developmental trend in social information use, but also children from non-Caucasian societies (most pronouncedly in Indonesia, but also in Kenya and Namibia). Moreover, the U-shaped developmental pattern with respect to the majority bias (Fig. 1b) is robust across societies and cannot be explained by an ethnic bias for both the majority and minority consisted of Caucasian children.

Note that within the developmental U-shaped pattern with respect to the majority bias, cultural variation could be identified by comparing preferences within age categories. For instance, the 4–6-year olds from Indonesia, Kenya and Zambia seem substantially less inclined to follow the majority than their counterparts from Brazil, the Central African Republic, Germany and Namibia (Fig. 2b). This cross-sectional detail corroborates the necessity to study ‘the social learning of social learning strategies’^38,39^. Indeed, our broader finding, revealing the culture-general notion of the U-shaped majority preference, highlights the importance of assessing ontogenetic trajectories for charting cultural variation.

In comparison to other animal species, humans show extraordinary variability across societies^1,2^. We propose that in order to apprehend human uniqueness, we need to understand the dynamic interplay between human ontogeny and the emergence of culture. An encompassing theory of the human mind must therefore consider the ways in which human psychology adapts to, and shapes, social and ecological contexts, as well as the universal foundations that enable this reciprocal interaction.

## Methods

### Sampling procedure and data handling

Nine societies were opportunistically sampled as part of a larger research endeavour of the Comparative Cognitive Anthropology research group of the Max Planck Institute for Evolutionary Anthropology (Leipzig, Germany) and the Max Planck Institute for Psycholinguistics (Nijmegen, the Netherlands) led by DBMH (2009–2013). At the respective research sites, subjects were opportunistically recruited (i.e., all available parents and respective children were approached for participation in the experiment), leading to a slightly unbalanced study sample (Supplementary Tables 1 and 2). At each field site, informed consent forms (in the local language) signed by the children’s parents, parental representatives, local authorities, community elders and/or teachers were obtained prior to testing the children. All study procedures were approved by the Max Planck Institute for Evolutionary Anthropology, Leipzig, Germany.

When conditions permitted, sessions were video-recorded for later scrutiny. All video-recorded sessions (80% of all sessions) were checked for (i) procedural adequacy, and (ii) corroboration of live-scored responses by two independent coders. Digression from the outlined procedure was judged in light of the a priori formulated inclusion criteria (Supplementary Table 3). Corroboration of the live-scored responses was optimal (100%).

### Participants

We tested 681 children (341 boys, 340 girls, age range 4–14 years) across nine societies based on availability at the respective field sites (Supplementary Notes 1). Prior to analysis, we formulated and applied inclusion criteria (Supplementary Table 3) after which we obtained a sample including 657 children (331 boys, 326 girls, age range 4–14 years). For reasons of suspected communication between participants during the experiment, we excluded all children from Pangkalan Bun (28 children, age range: 6–7 years) from the analyses (see Supplementary Notes 2 for the script used to detect out-of-scale correlation between successive responses). We furthermore excluded all children from Samoa (24 children, age range: 5–8 years) due to the impossibility of obtaining reliable model estimates (see Supplementary Notes 2 and Supplementary Fig. 3 for the model stability results). Excluding Samoa did not qualitatively change the results (see Supplementary Notes). The final sample comprised 605 children (306 boys, 299 girls, age range: 4–14 years, mean age ± SD: 8.11 ± 2.50 years) from seven different societies [Brazil, Central African Republic (BaAka), Germany, Indonesia, Namibia (≠Akhoe Hai//om), Kenya (Samburu) and Zambia (Bemba)]. We refrained from applying subjective cutoffs with respect to the number of participants per age or culture. Generalised linear models are robust against unbalanced designs if model stability measures are taken into account.

### Design

We used a one-shot design to maximise data independency. Instructions to the children were standardised and given in the local language by trained experimenters. The demonstrations were counterbalanced in terms of order of majority and minority demonstrations, and in terms of which coloured pipes were used by the demonstrators.

### Experimental procedure

**Fig. 3:**
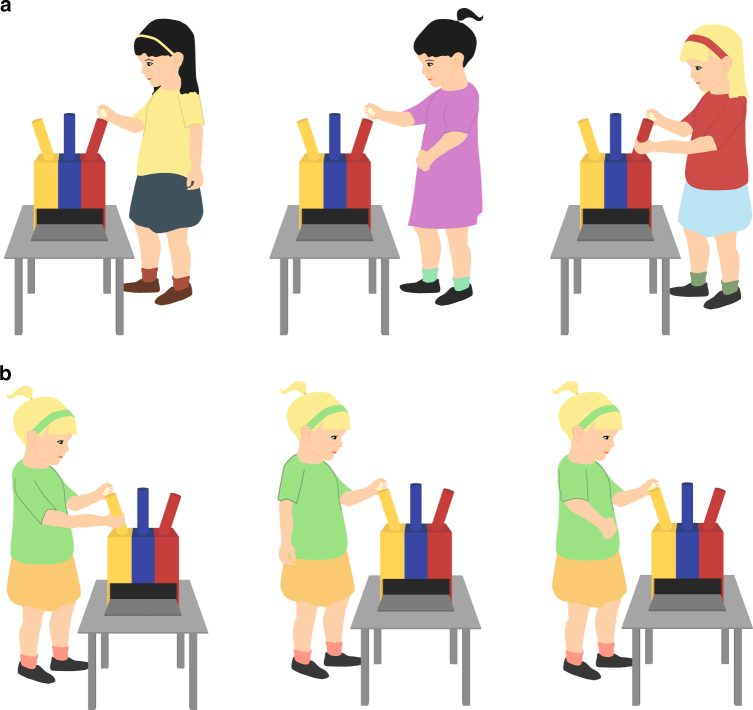
Experimental set-up. Illustration of the apparatus, including the **a** majority and **b** minority demonstrations. Upon dropping the ball into the pipe, a reward was automatically released from the apparatus

Children, who were naive with respect to the task, were called into a classroom one after another to partake in the experiment. Nothing at this stage was revealed about the aim of the experiment itself. Children received the following instructions in their local language: 'I have brought a box that has three openings. If one throws a ball in the correct hole, a toy is delivered from the box. I show you a movie now in which four different children will show you how to use it. Thereafter, you can use it yourself. Watch closely!'. Children then watched the video demonstrations individually. The video depicted four peers demonstrating how to use the box (Fig. 3). The demonstrators were the same gender as the observer. The video demonstrations during the experiment were non-verbal, yet a supporting narration in the child’s local language was presented by the experimenter in sequence according to counterbalance condition: Minority: 'This is [x = pre-determined locally appropriate name], (s)he uses the yellow hole. This is [x] again, (s)he uses the yellow hole again. This is again [x], (s)he uses the yellow hole again' (i.e., three times same demonstrator, same hole).

Majority: 'This is [y = different pre-determined locally appropriate name], (s)he uses the blue hole. This is [z = different pre-determined locally appropriate name], (s)he uses the blue hole. This is [a = different pre-determined locally appropriate name], (s)he uses the blue hole'. After the video demonstration, children received a final instruction in their local language: 'Now you can use the box. Beware; you only have this one ball'. The 'choice-box' contained three differently coloured pipes. If a ball was dropped in any one of the pipes, a reward was automatically released from the box. After the child had used the 'choice-box' and received the reward, he/she was thanked and escorted to another room (or outside) in order to minimise the chance for them to communicate with the remaining naive children.

### Statistical methods

We tested children’s reliance on social information (vs. innovation), and children’s preference for the majority (vs. the minority). Both tests were statistically approached using generalised linear models with binomial error structure and logit link function^39^, and fitted with the function 'glm' in R (version 3.4.1)^40^. First, we established the significance of our full model^41^ (comprising 'culture', 'age', 'sex', and, to allow for fluctuating age trends and cultural variation, the polynomial 'age squared' and the interactions of culture with the age variables, respectively) by comparing it to our null model (comprising only 'order of demonstrations') with a likelihood ratio test^42^ (R function 'anova': *χ*^2^ = 35.6, df = 21, *p* = 0.024). Second, we assessed parameter contribution to explaining variation in the dependent variable [(i) social information use vs. innovation, and (ii) majority vs. minority preference) by comparing full with respective reduced (i.e., only lacking the variable under scrutiny) models using likelihood ratio tests^42,43^. The variable 'age' was *z*-transformed to a mean of 0 and a standard deviation of 1. Specifically, in our first model (*n* = 605), the outcome variable was 'yes/no followed demonstrations', with '1' indicating the majority or minority response, and '0' the third, non-demonstrated option. In our second model, we selected only those children that followed one of the social demonstrations (response '1' in the first model; *n* = 475), and tested whether the majority was favoured over the minority by having our outcome variable being 'yes/no followed the majority', with '1' indicating the majority response, and '0' indicating the minority response. Odds ratios for significant dichotomous parameters were obtained by exponentiation of the respective model estimates^44^. Children’s responses were only used if children’s comprehension of the task was judged as valid by the experimenter onsite as well as two independent video-coders (Supplementary Table 3).

### Data availability

The data were collected based on availability of subjects at the field sites. No selection criteria were applied in recruiting subjects other than age and local residence. The data sets generated and analysed during the current study are available in the Open Science Framework repository (https://osf.io/m3ub7).

## Electronic supplementary material

Supplementary Information

Peer Review File
