# Supplementary material for: The development of human social learning across seven societies
Source: Nat Commun. 2018 May 25;9:2076. doi: 10.1038/s41467-018-04468-2 (PMC5970179; doi:10.1038/s41467-018-04468-2)
Supplement: Supplementary file 1 — Supplementary Information [file 41467_2018_4468_MOESM1_ESM.pdf]

Supplementary Information:

The development of human social learning across seven societies

van Leeuwen et al.

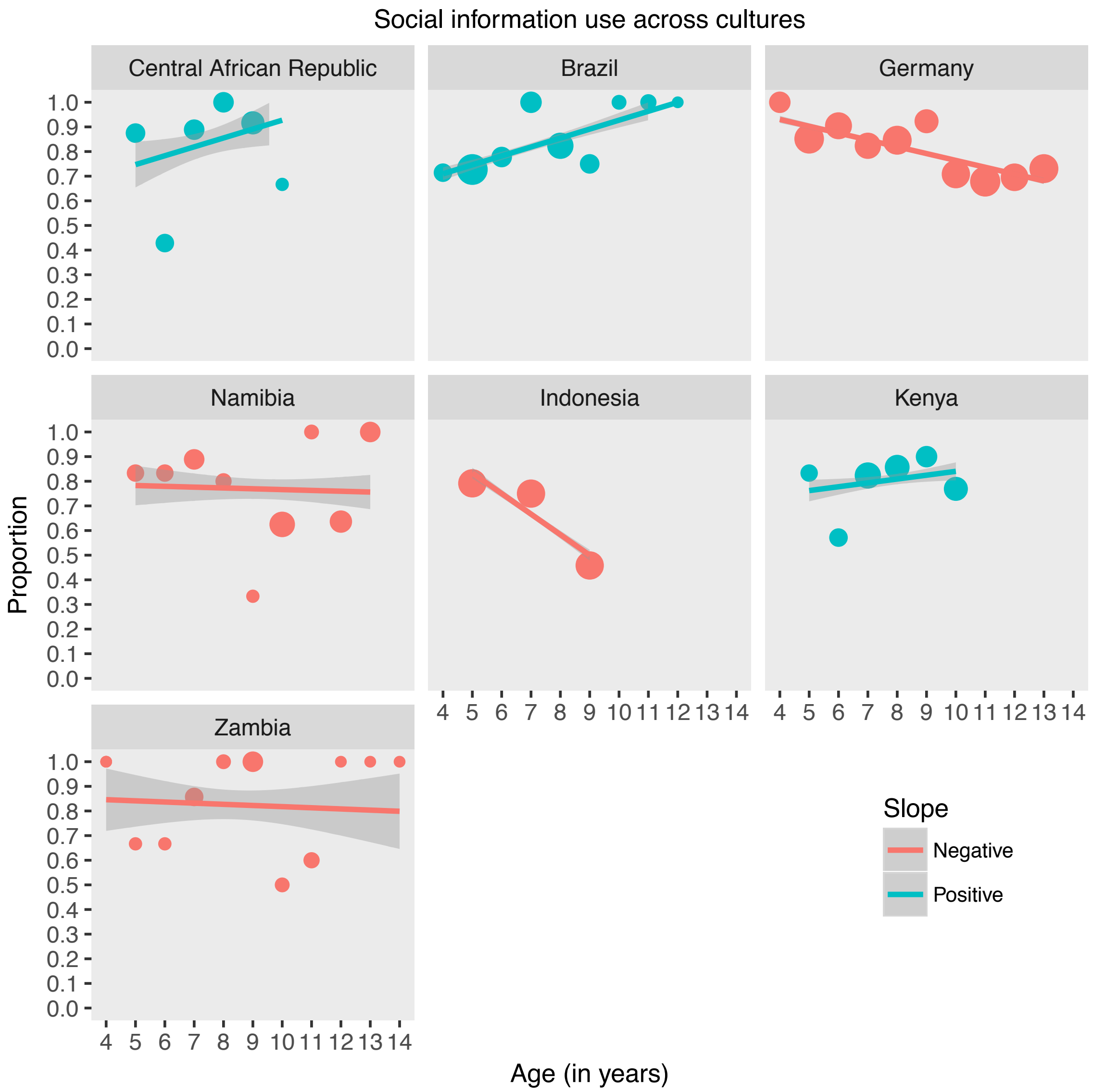

Supplementary Figure 1: The ontogeny of social information use (versus innovation) across cultures. The colours indicate negative (red) or positive (green) slopes. The shaded area around the lines represents the 95% confidence interval. The relative size of the dots is proportional to the number of observations. Dot size is proportional to the number of observations in ratio 1:5, with minimum and maximum number of observations per dot being 1 and 33, respectively.

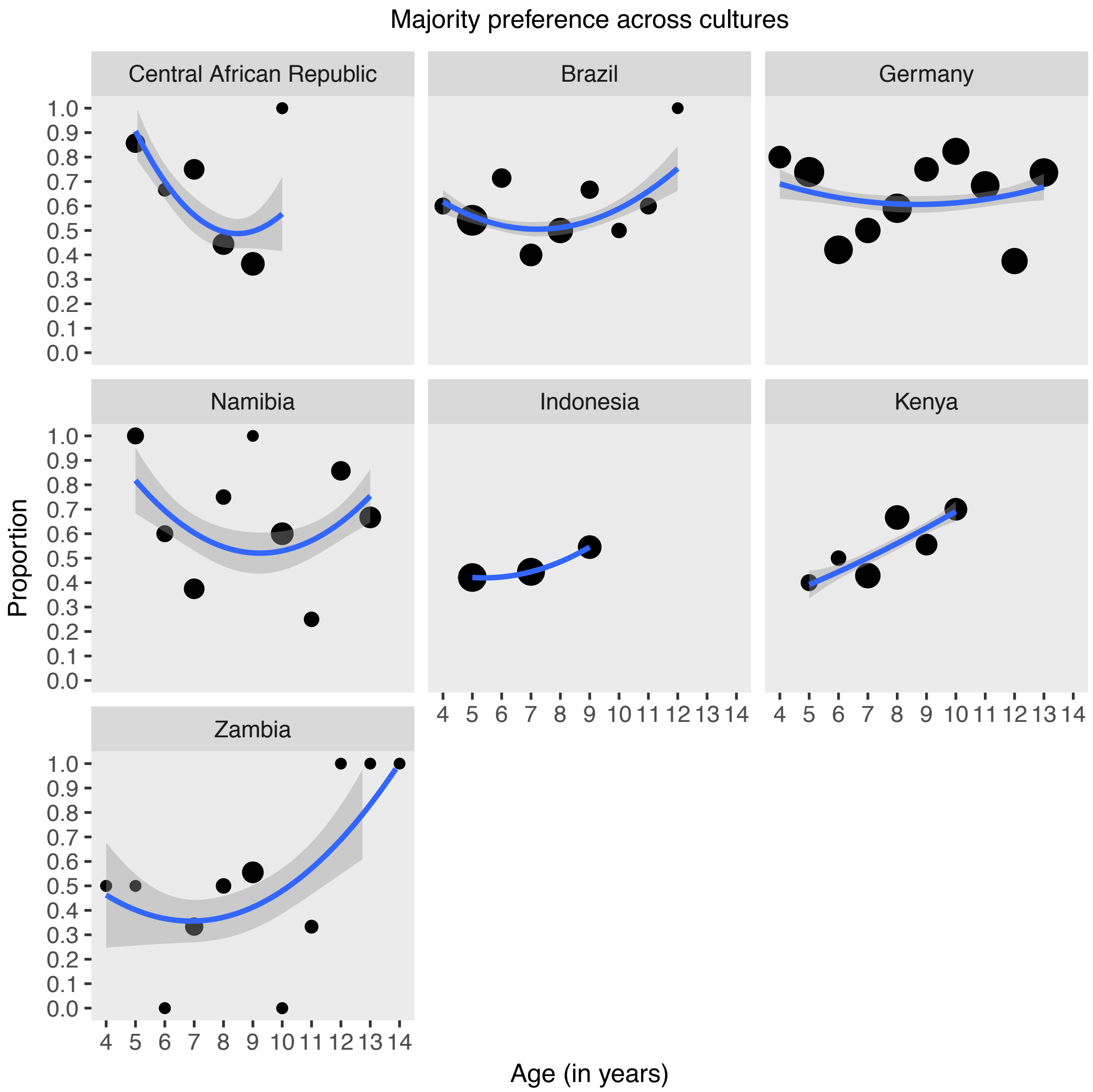

Supplementary Figure 2: The ontogeny of majority (versus minority) preference across cultures. The shaded area around the lines represents the 95% confidence interval. The relative size of the dots is proportional to the number of observations. Dot size is proportional to the number of observations in ratio 1:5, with minimum and maximum number of observations per dot being 1 and 24, respectively.

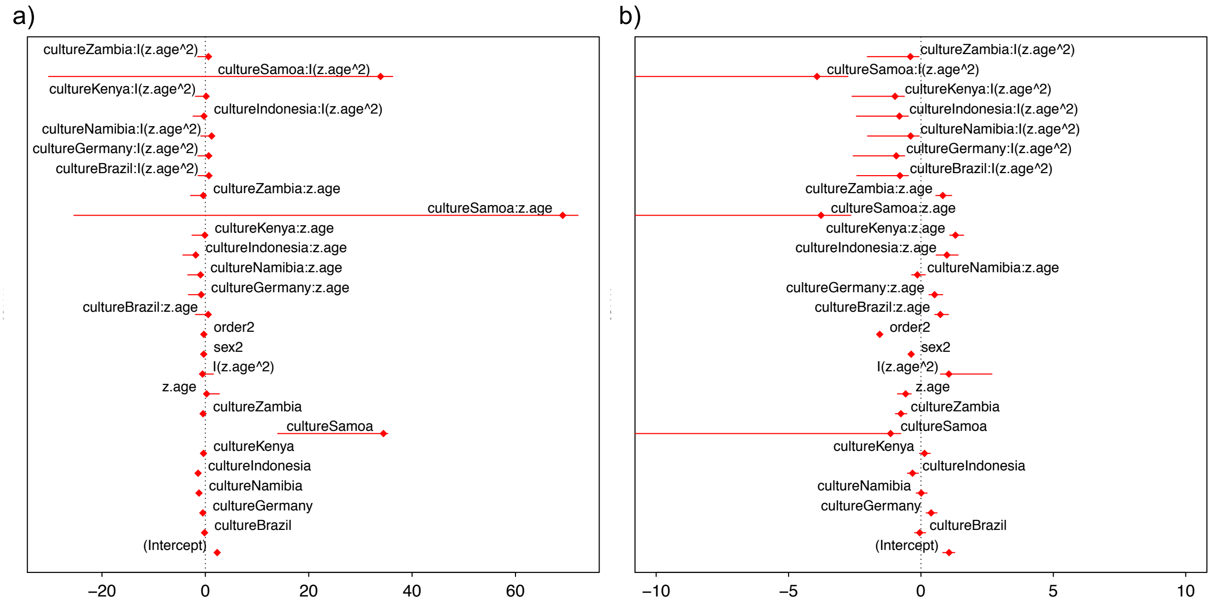

Supplementary Figure 3: Model Stability. Model stability indices based on subject omissions (n-1) from the full model for a) social information use (versus innovation), and b) majority (versus minority) preference. For each term in the model, a red horizontal line depicts the range of its model estimate across all n-1 runs. In comparison to the other terms, all variables including Samoa show extreme variability, indicating that our models cannot reliably estimate values for Samoa.

Supplementary Table 1. Subjects in sample for analysing social information use (n=605)

Age	Brazil	Central African Republic	Germany	Indonesia	Kenya	Namibia	Zambia
**4**	7	0	10	0	0	0	2
**5**	33	8	27	24	6	6	3
**6**	9	7	21	0	7	6	3
**7**	10	9	17	24	17	9	7
**8**	17	9	26	0	14	5	4
**9**	8	12	13	24	10	3	9
**10**	4	3	24	0	13	16	4
**11**	5	0	28	0	0	4	5
**12**	1	0	23	0	0	11	1
**13**	0	0	26	0	0	9	1
**14**	0	0	0	0	0	0	1
**Total**	**94**	**48**	**215**	**72**	**67**	**69**	**40**

Supplementary Table 2. Subjects in sample for analysing majority preference (n=475)

Age	Brazil	Central African Republic	Germany	Indonesia	Kenya	Namibia	Zambia
**4**	5	0	10	0	0	0	2
**5**	24	7	23	19	5	5	2
**6**	7	3	19	0	4	5	2
**7**	10	8	14	18	14	8	6
**8**	14	9	22	0	12	4	4
**9**	6	11	12	11	9	1	9
**10**	4	2	17	0	10	10	2
**11**	5	0	19	0	0	4	3
**12**	1	0	16	0	0	7	1
**13**	0	0	19	0	0	9	1
**14**	0	0	0	0	0	0	1
**Total**	**76**	**40**	**171**	**48**	**54**	**53**	**33**

Supplementary Table 3. In- and exclusion criteria applied on video recordings.

**drop out** (n=6)	Subject was helped/led by instructor
**drop out** (n=2)	Previous subject possibly told the subject a colour to use/Subject was instructed by others
**drop out** (n=2)	Instructor points on a special hole right before subject enters the ball
**drop out** (n=3)	Instructor points on all holes only once right before subject throws the ball
**drop out** (n=3)	Subject was not able to access all holes of the box
**drop out** (n=3)	Subject seemed not to understand the instructions
**drop out** (n=2)	Subject did not receive the instructions, only saw the video
**drop out** (n=3)	Obstructed view: instructor may have led subject to an opening because she/he raised subject's arm in which it held the ball
**keep**	Wrong mention by experimenter while showing the video (but corrects himself immediately)
**keep**	Subject looks at experimenter for help when entering the ball.
**keep**	Subject needed help to access the box
**keep**	Subject very hesitant, repeatedly looking at the instructor for help, throws ball after very long time
**keep**	Subject very hesitant, repeatedly looking at the instructor for help, video shown again
**keep**	Interruption by another person while showing the video
**keep**	Toy does not come out when subject has entered the ball
**keep**	Instructor nods when child holds ball over opening and looks back to the instructor for agreement before entering the ball
**keep**	Interruption by another person after showing the video, video shown again
**keep**	Toy was put into the box when subject was present in the room
**keep**	You cannot see in which hole the subject threw the ball on video
**keep**	Not recorded, SD Card was full

Supplementary Note 1

Fieldsite Information

Country: Brazil (*Brazilian*)[1]

The research was conducted in the town of Soure on the Island of Marajó in northern Brazil, a town home to ±23,000 inhabitants. The archipelago of Marajó covers an area of approximately 50,000 km^2 at the mouth of the Amazon, in the northern Brazilian state of Pará. Marajó is the largest fluvial-marine island in the world. The island vegetation is split between the terra firme, floodplain and flooded forests of the west and the savannah and mangroves to the east, where the present study was based. The year is also split between rainy and dry seasons (January-June and July-December, respectively).

Soure is one of seven municipalities within the island's Ararí micro-region. According to the 2010 census of the Brazilian Institute for Geography and Statistics (IBGE), the municipal population of Soure was 23,001 (including both the town inhabitants and all rural populations in its 3,517 km^2 territory). Access from the mainland to the nearest main port, Cametá, is via a 3-hour boat journey across the Bay of Marajó from the state's capital, Belém. Buffalo and cattle ranching as well as timber and palm extraction drive the local wage economy. Fishing is also an important economic activity throughout the archipelago.

Socioeconomically, however, the region's infrastructure, public services and facilities are underdeveloped and it remains isolated from the progressive urban centres of the mainland. Average monthly salary in Soure was 644 Brazilian Reais (approx. 195 USD) as compared with 1512 Brazilian Reais in the state capital of Belém, and just under half of the population were recorded as below the poverty line (48.2%).

The municipality has 12 pre-schools, 22 primary schools, and 3 middle schools. The Federal University of Pará also has a presence in the town, offering courses in higher education. Figures record 62% of the population as having completed primary education.

Children typically live in nuclear or extended family households and, when not at school, are engaged in play and leisure activities for the most part.

Country: Central African Republic (*BaAka*)[2–4]

The research was conducted in a village (±450 inhabitants) in the Dzanga-Sangha Reserve in the south-west of the Central African Republic. The BaAka (also called Bayaka or Aka) live as traditional foragers in the tropical rainforest areas of southern Central African Republic and northern Republic of Congo. They speak Yaka, a distinct Bantu language. Hunting and gathering still make up a significant part of their subsistence activities, for example, the BaAka in the present community hunt a variety of small and large mammals with nets, spears and cross-bows, and collect forest products such as honey, insects, mushrooms and a variety of plants. Forest products and temporary labor are partially traded for agricultural products with neighboring Bantu farmers. BaAka families are typically mobile: they live in the villages together with the sedentary Bantu farmers parts of the year but they also spend several weeks or months in small, temporary camps in the forest.

As typical for many hunting and gathering groups, BaAka society is characterized by a highly egalitarian social structure without formal leaderships and only a few status roles.

When families live in the villages, some children visit the local school in which they are taught in Sango and French (national languages of the Central African Republic), but not in Yaka, their mother tongue. School attendance among BaAka children is rather inconsistent, and often problematic due to the subsistence activities and mobility of their families. Most of the younger children spend their time playing around camps, while older children occasionally join adults on foraging trips to the forest.

Country: Germany (*German*)[5,6]

The research was conducted in “Leipzig”, a city in the eastern part of Germany, home to ±580.000 inhabitants. Germany is an industrialized parliamentary republic in Europe. It has a temperate seasonal climate and 82 million inhabitants. Leipzig is Germany's tenth most populous city with a population of almost 600.000 inhabitants. The German language is a member of the Germanic subfamily of the Indo-European language family and the mother tongue of about 95 million people worldwide. Germany has a social market economy and the largest national economy in Europe. Christianity is the largest religion in Germany (+/- 65% of the total population). Optional preschool education is provided for children between one and six years of age, after which school attendance is compulsory for at least nine years.

Country: Indonesia (*Javanese, Bornean*)[7]

The research was conducted in a school in Indonesia’s capital city Jakarta, a city on the Java island, home to ±10.3 million inhabitants, and in Pankalan Bun, a city in Central Kalimantan (Kalimantan Tengah) province on Borneo, with around 200,000 inhabitants.

Indonesia is the world’s largest archipelago with more than 17,000 islands, hundreds of distinct ethnic and linguistic groups, and a population of over 261 million. Indonesia is the most populous Muslim-majority country in the world. Located in the north west of Java, Jakarta is a fast growing megalopolis and the centre of Indonesian economics, politics and culture.

Javanese children all went to the same private fee-paying school established in 1996 in a middle to upper class urban area of Jakarta. Private schools only comprise 7% of the schools in Indonesia ("World Bank and Education in Indonesia". World Bank. 1 September 2014). Known as a “national plus school”, this school is nationally registered as an Indonesian private school but with a dual language curriculum in Bahasa Indonesia and English and a multi-age system in which classes are grouped according to the student's age.

Bornean children were tested while visiting an environmental education centre run by a local NGO (Yayorin).

While Indonesia is extremely diverse and undergoing fast development, the society as a whole is frequently described as hierarchical and collectivist. Power disparity based on position is high and obedience is expected from those lower in power. Communication is often indirect and negative feedback avoided. Indonesian society in general has a strong hierarchical framework in which individuals show loyalty to their groups. Indonesia is known as a collectivist society with strong respect and obedience shown by children to their parents and elderly family members usually cared for at home into old age.

Country: Kenya (*Samburu*)[8]

The research was conducted in a small Samburu community (±400 inhabitants) in a remote rural area north of Maralal (Samburu District, Kenya). The Samburu are pastoralists who live mainly in the arid lands of north-central Kenya. Being closely related to the Maasai, they speak a dialect of Maa (a Nilo-Saharan language). Traditionally, Samburu families own livestock (e.g., cattle, goats, sheep, camels) which provides them with milk and occasionally meat. Additionally, nowadays, some families in some communities try seasonal gardening around their homesteads and some men engage in wage work. Although a part of the Samburu population is still nomadic and seasonally moves with the cattle to different pastures, for the most part families in the present community are sedentary and stay in the village throughout the year.

Many aspects of Samburu society are determined by a 'gerontocracy' - a strict age-based hierarchy. Group elders, in particular men, make most of the important decisions within families and communities and many personal rights and obligations (within and outside the family) depend on a person's status. For example, young men typically go through a long stage of bachelorhood (called 'murranhood') during which cultural rules suspend them from

households and the rest of the community. Only around 30 years of age, when they have acquired own livestock and the elders decide, they are allowed to marry and start their own families.

Children who participated in the study all went to the community's primary school or the adjacent preschool in which they were taught in Kiswahili, English and sometimes in their mother tongue. Outside of school, older children help with adult activities such as caring for animals and household chores.

Country: Namibia (*≠Akhoe Hai//om*)[9]

The research was conducted in a small community in northern Namibia called "Farm 6", Mangetti West, home to ±200 ≠Akhoe Hai//om people. ≠Akhoe Hai//om are traditionally hunter-gatherers, living in the Northern Namibian dry-lands between the Etosha salt-pan and the Namibian-Angolan border. In the last decades, due to political and economic marginalization, many aspects of their traditional culture have been lost. However, while gardening and wage-work have increased in frequency and relevancy, hunting and gathering are still practiced regularly in many ≠Akhoe Hai//om communities. Other examples of retained traditional practices are healing trance dances, the traditional kinship and naming system and a landscape-based system for orientation.

The language, ≠Akhoe Hai//om, is part of the Khoekhoe cluster of languages and employs click sounds that are unfamiliar to speakers of Indo-European languages. Although largely sedentary due to the privatization of land in Namibia, the ≠Akhoe Hai//om of "Farm 6" still frequently move between settlements for long periods of time.

The social structure of the ≠Akhoe Hai//om is egalitarian, as is considered typical for Hunter-Gatherer communities: Adults do not typically wield the authority to speak on behalf of another adult within the community. Decisions concerning the community as a whole are based on consensus. Generally speaking, in ≠Akhoe Hai//om communities, differences in

social status, be it based on skill, experience or possessions, are de-emphasized, often even socially sanctioned against.

Children spend most of their time in peer groups engaging in play. To a limited amount, they are also involved in household chores such as collecting fire-wood or minding younger siblings. Since 2005, children have access to a school offering grades 1 through 6. Approximately 50% of children attend school.

Country: Samoa (*Samoan*)

The research was conducted in a school in Safotu, a village on the north coast of the island of Savai'i, home to ±1400 inhabitants. The independent state of Samoa consists of two main islands of volcanic origin and a couple of smaller islets, predominantly uninhabited. It is located in the Polynesian region south of the equator, about 3000 km north-east of New Zealand. Samoa is sometimes still referred to as Western Samoa in order to distinguish it from American Samoa which is situated further east and is an unincorporated part of the United States of America.

Samoa's population exceeded 195,000 inhabitants in 2016[10], with about 43,000 people living on the island of Savai'i. Apart from the capital Apia on the island Upolu, communities mainly live in villages on flat land along the coastline. The spatial structure of these villages has remained much the same until today. Private houses are organized around a central common place (*malae*), which suits as a place for gatherings, festivities and such.

Samoan society is organized in large parts by a chiefly system (*Fa'amatai*). The chiefly title *matai* is given to family members with recognized ability within the extended family (*aiga*). Multiple *matai* can be appointed within *aiga*. The *matai* looks after the wellbeing of the family clan. Matters of communal interest are discussed by a village council (*fono*) that consists of the *matai* of all families belonging to the village. *Fa'amatai* plays a

major role even in modern Samoan politics as only *matai* are endowed with the right to hold political office.

About 99% Samoans consider themselves to be of Christian belief[11]. Religiosity is deeply rooted within and has a significant influence on people’s lives.

Social culture within families is formed by respect for the elders. Children are expected to take an active role in the family duties from around the age of five and comply with the demands and wishes of the elders. Their following traditional Samoan customs and Christian practices are of great importance for the family’s reputation within the communities.

Country: Zambia (*Bemba*)[12,13]

The research was conducted in a small community in northern Zambia at the Chimfunshi Wildlife Orphanage, Chingola, home to ±250 Bemba people. The Bemba are traditionally subsistence farmers. Typically, the Bemba live in rural villages organized around a number of extended families. The extended family is a cooperative work group that shares food, gifts, money, and other material items. Within the extended family system, a person usually has several "mothers," several "fathers," and many "sons" and "daughters."

The villages generally have thirty to fifty houses (“huts” made of wattle and daub – woven rods and twigs plastered with clay and mud – and thatched roofs). Because the main area of the Bemba distribution consisting of bush, scrub and low trees, the Bemba have been referred to as a forest people (Miombo forest, also typical of the area where this study was conducted). Through their practicing of *Chitemene* – a system in which crops are grown in the ash produced by burning wood from a cleared forest area – villages typically shifted location when the soil got depleted.

The language – the Bemba – belongs to the Bantu language family. Approximately seventy-two tribes live in Zambia, roughly divided in seven ethnic groups: Nyanga, Lozi, Lunda, Kaonde, Bemba, Luvale, Tonga. Most languages are not mutually comprehensible.

The social structure of Bemba societies is strongly regulated by chiefdoms, with one paramount chief commanding lesser chiefs (all drawn from the one Royal Crocodile Clan), who in turn appoints village headmen. These village headmen run villages and mediates in conflicts and access to land (i.e., for farming). Village-life still comprises traditional practices and beliefs, like supernatural medicine, ancestors' spirituality, and black magic.

Children learn household, agricultural, and hunting skills from their mother or her relatives, although the father may be involved. Children have freedom and autonomy but must respect their elders. Although the practice has declined in recent years, initiation *(ichisungu)* at puberty teaches girls duties toward their households and husbands. There are no equivalent male initiation ceremonies. Children generally attend school, although in remote areas like Chimfunshi, this is not trivial (grades 1-6 are offered since 2007; grades 7-10 since 2015).

Supplementary Note 2

Analyses applied to optimize reliability of our findings

Given the opportunistic nature of our data-collection procedure, in order to establish adequate reliability of our findings, we applied two established techniques to our data, *i*) a Runs Test for Detecting Non-Randomness[14], and *ii*) a Model Stability assessment[15]. The first technique was meant to obviate any responses affected by information from already tested children, the second technique represents a procedure to identify unstable variables in our model.

Assessing the likelihood that naïve children were informed by tested children

Despite procedural countermeasures, children might have found a way to communicate with each other about the "correct" answer, likely expressed in terms of a particular colour. With the following runs.test script (kindly provided by Roger Mundry), we tested whether certain series of the same-colour responses occurred suspiciously frequently based on the relative prevalence of the respective colour in the entire set of responses, taking into account the fact that the video-demonstrations were counterbalanced across the three colours.

```
runs.test<-function(x, n.perm=1000){
    x=as.factor(x)
    n=length(x)
    test.stat=rep(NA, n.perm)
    test.stat[1]=sum(diag(table(x[-n], x[-1])))
    for(i in 2:n.perm){
        x=sample(x, n, replace=F)
        test.stat[i]=sum(diag(table(x[-n], x[-1])))
    }
    return(list(test.stat=test.stat[1],
 P.too.many=mean(test.stat>=test.stat[1]),
 P.too.few=mean(test.stat<=test.stat[1])))
}
```

In other words, successive same-colour responses are possible, but when the same colour is chosen many times in a row, where "many times" is taken relative to the total number of

responses of each colour, then the "correct" colour (i.e., the one that produced a toy for the already tested subject) may have been communicated by the tested to the naïve subjects.

The *p*-values concerning the test of "too many" same-colour responses in a row for all societies but Pangkalan Bun were non-significant ($p > 0.2$). Data from Pangkalan Bun ($p < 0.01$) were consequently classified as "unreliable" and discarded from the study.

Model Stability – Identifying unstable variables

Given the unbalanced study sample and the resulting fact that the age-ranges of the children tested across the different societies did not overlap perfectly, in order to avoid spurious results, we checked for model stability by assessing the ranges of model estimates based on n-1 model runs with successive subject omissions, and included only those variables in the final model analysis that showed a reasonable extent of robustness[15] (Supplementary Figure 3). Both in the analysis of social information use and majority preference, Samoa emerged as a highly unstable variable in all its appearances (i.e., as part of the interactions and as main effect).

<u>Model a)</u>: *social information use (y/n) ~ culture*(age+age²)+sex+order*

<u>Model b)</u>: *majority preference (y/n) ~ culture*(age+age²)+sex+order*

In part, this instability of estimates for Samoa may be the result of the fact that the Samoan sample was the smallest sample of all the respective societies (i.e., $n = 24$). In any case, based on the finding that the Samoan estimates differ tremendously across models differing only by one subject (Supplementary Figure 3), we opted for omitting the Samoan sample from our final analysis. Nonetheless, in the following section, we additionally report the results including the Samoan sample. In sum, all results remain qualitatively unchanged. For the sake of completeness, we also analysed the entire data-set (n=657), thus including the data from Pangkalan Bun, finding that all results remain qualitatively unchanged.

Supplementary References

1. Schaan, D. P. in *The Handbook of South American Archaeology* 339–357 (Springer New York, 2008).
2. Hewlett, B. S. *Intimate fathers: the nature and context of Aka pygmy paternal infant care*. (University of Michigan Press, 1992).
3. Noss, A. J. The Economic Importance of Communal Net Hunting Among the BaAka of the Central African Republic. *Hum. Ecol.* **25,** 71–89 (1997).
4. Bahuchet, S. Food sharing among the Pygmies of Central Africa. *Afr. Study Monogr.* **11,** 27–53 (1990).
5. Haspelmath, M., Dryer, M. S., Gil, D. & Comrie, B. *World atlas of language structures*. (Oxford University Press, 2005).
6. Buse, D. K. & Doerr, J. C. *Modern Germany : an encyclopedia of history, people, and culture, 1871-1990*. (Garland Pub, 1998).
7. Hofstede, G., Hofstede, G. J. & Minkov, M. *Cultures and Organizations, Software of the Mind: Intercultural Cooperation and its Importance for Survival*. (McGraw Hill, 2010).
8. Spencer, P. *The Samburu: A Study of Gerontocracy in a Nomadic Tribe*. (Routledge, 2004).
9. Widlok, T. *Living on Mangetti: 'Bushman' Autonomy and Namibian Independence*. (Oxford University Press, 1999).
10. https://data.worldbank.org/country/samoa.
11. https://www.cia.gov/library/publications/the-world-factbook/geos/ws.html.
12. Ferguson, J. *Expectations of modernity: myths and meanings of urban life on the Zambian Copperbelt*. (University of California Press, 1999).
13. Roberts, A. *A history of the Bemba: political growth and change in north-eastern Zambia before 1900*. (University of Wisconsin Press, 1973).

14. Bradley, J. V. *Distribution-free statistical tests*. (Prentice-Hall, 1968).

15. Nieuwenhuis, R., te Grotenhuis, M. & Pelzer, B. Influence.ME: tools for detecting influential data in mixed effects models. *R J.* **4,** 38–47 (2012).
